# Trends and determinants of early initiation of breastfeeding and exclusive breastfeeding in Ethiopia from 2000 to 2016

**DOI:** 10.1186/s13006-019-0234-9

**Published:** 2019-09-11

**Authors:** Kedir Y. Ahmed, Andrew Page, Amit Arora, Felix Akpojene Ogbo

**Affiliations:** 1 Translational Health Research Institute, Western Sydney University, Campbelltown Campus, Locked Bag 1797, Penrith, NSW 2571 Australia; 2 College of Medicine and Health Sciences, Samara University, PO Box: 132, Samara, Ethiopia; 3 School of Science and Health, Western Sydney University, Campbelltown Campus, Locked Bag 1797, Penrith, NSW 2571 Australia; 4 Oral Health Services, Sydney Local Health District and Sydney Dental Hospital, NSW Health, Sydney, Australia; 5 Discipline of Child and Adolescent Health, Sydney Medical School, Faculty of Medicine and Health, The University of Sydney, Weastmead, NSW Australia; 6 General Practice Unit, Prescot Specialist Medical Centre Makurdi, Welfare Quarters, Makurdi, Benue State 972261 Nigeria

**Keywords:** Early initiation of breastfeeding, Timely initiation of breastfeeding, Exclusive breastfeeding, Infant and young child feeding, Ethiopia

## Abstract

**Background:**

At the national level in Ethiopia, there is limited knowledge of trends and factors associated with early initiation of breastfeeding and exclusive breastfeeding (EBF), particularly during the Millenium Development Goal (MDG) era (2000–2015). The study aimed to examine the trends and determinants of early initiation of breastfeeding and EBF in Ethiopia between 2000 and 2016.

**Methods:**

Using the Ethiopia Demographic and Health Survey (EDHS) data for the years: 2000 (*n* = 3680), 2005 (*n* = 3528), 2011 (*n* = 4037) and 2016 (*n* = 3861), trends in early initiation of breastfeeding and EBF were estimated. Multivariate logistic regression models that adjusted for confounders, sampling weight, clustering and stratification were used to examine the association between socioeconomic, demographic, health service and community level factors with early initiation of breastfeeding and EBF from 2000 to 2016.

**Results:**

The prevalence of early initiation of breastfeeding increased from 48.8% in 2000 to 75.7% in 2016 in Ethiopia. Improvement in EBF prevalence was not statistically significant (from 54.5% in 2000 to 59.9% in 2016). Over the study period, informal maternal employment (Adjusted Odds Ratio [aOR] 0.75; 95% Confidence Interval [CI] 0.68, 0.83), frequent antenatal care visits (aOR 0.74; 95% CI 0.65, 0.85), and cesarean birthing (aOR 0.22; 95% CI 0.17, 0.30) were associated with delayed initiation of breastfeeding. Birthing in the health facility (aOR 1.35; 95% CI 1.05, 1.75) and residing in the metropolis region (aOR 1.95; 95% CI 1.65, 2.32) were associated with timely initiation of breastfeeding. In a similar period, informally employed mothers (aOR 1.37; 95% CI 1.15, 1.63) and those with six or more family size (aOR 1.46; 95% CI 1.10, 1.93) were more likely to exclusively breastfeed their babies.

**Conclusion:**

Early initiation of breastfeeding improved in Ethiopia during the MDG era but it is still below the national target; progress in EBF remained slow. To improve breastfeeding outcomes and meet the global breastfeeding targets in Ethiopia, infant feeding efforts should focus on improving key modifiable factors, including place and mode of birthing and socioeconomic status of mothers.

## Background

Optimal breastfeeding is the best source of nutrition for the newborn and the cornerstone for establishing a healthy growth and development for children [1, 2]. Optimal breastfeeding is beneficial to the infant, the mother, the household and the community. Optimally breastfed babies are less likely to develop childhood infections (such as diarrhoea, otitis media and pneumonia) [2–4]. In later life, optimal breastfeeding is associated with a reduced risk of overweight or obesity and type 2 diabetes, and a higher cognitive function has been demonstrated among children who were exclusively breastfed for up to 6 months [1]. Mothers who optimally breastfeed have a reduced incidence of type 2 diabetes mellitus, breast and ovarian cancers [5–7]. They are also more likely to have improved birth spacing and social-emotional interaction with their babies [8]. At the community level, good nutrition of children contributes to improved human capital through a reduction in expenses on infant formula and increase opportunities for a more sustainable future [9].

In view of the benefits of optimal breastfeeding, the World Health Organization and the United Nations Children’s Fund (WHO/UNICEF) [10] recommend early initiation of breastfeeding within the first hour of birth and exclusive breastfeeding (EBF) for the first 6 months of life, as well as continued breastfeeding until the child is 2 years of age [11]. However, current evidence suggests that approximately 38% of infants are exclusively breastfed worldwide [5], while timely initiation of breastfeeding varied globally, higher in high-income countries than low and middle income nations [1]. More recently, the United Nations renewed its commitment to improving child nutrition (including breastfeeding outcomes) worldwide through the United Nations Decade of Action on Nutrition (2016–2025) [12] and the Sustainable Development Goal (SDG-2.2) of ending all forms of malnutrition [13].

In low and middle income countries (LMIC, including Ethiopia), where access to clean water, adequate sanitation, and basic health and social services are often limited, the effects of suboptimal breastfeeding are even more prominent [9, 14]. For example, a recent study from Nigeria indicated that an estimated 23% of diarrhoea deaths among children under five were attributable to suboptimal breastfeeding in 2016 [15]. In Ethiopia, many subnational reports indicated that early initiation of breastfeeding (66.5%) and EBF (60.1%) [16] were well below the Ethiopian Health Sector Transformation Plan (HSTP) target of 90 and 72%, respectively [17]. Additionally, a systematic review of mostly subnational studies reported that government employment, having formal education, being knowledgeable, receiving guidance and counselling from health care workers, giving birth at a health facility and vaginal delivery were common factors associated with a higher practice of early initiation of breastfeeding and EBF in Ethiopia [16]. Although useful, these studies have the following limitations: (i) generalizability of the results due to increased heterogeneity of the study methodology, and under or over representation of some geographical regions [18–21]; (ii) trends in early initiation of breastfeeding and EBF were not examined to guide current and future efforts; (iii) limitations in the statistical analyses (such as adjustment for confounders and ignoring hierarchical nature of the data) [22, 23].

Understanding where progress has been made, where to allocate additional resources and who to specifically target to increase breastfeeding outcomes can provide insights into opportunities for scale-up and/or refinement of breastfeeding programs in Ethiopia. Such information would be useful to Ethiopian policy decision-makers and public health practitioners in providing specific policy interventions given the revitalized global efforts to improve child nutrition and end all forms of malnutrition. Accordingly, the present study aimed to examine the trends and determinants of early initiation of breastfeeding and EBF in Ethiopia between 2000 and 2016.

## Methods

### Data sources

This study was based on four rounds of Ethiopia Demographic and Health Survey (EDHS) data for the years 2000 (*n* = 3680), 2005 (*n* = 3528), 2011 (*n* = 4037), and 2016 (*n* = 3861). The surveys were implemented by the Central Statistical Agency (CSA) and Inner City Fund (ICF) international, and funded by the United States Agency for International Development, and the Government of Ethiopia [24–27]. The EDHS survey used a two-stage stratified cluster sampling technique to select the study participants. In stage one, after each administrative region was stratified into urban and rural strata, Enumeration Areas (EAs) were selected using a probability proportional to EA size. In stage two, a household listing operation was carried out in all of the selected EAs and a fixed number of households from each EA were selected [24–27]. All women aged 15–49 years who were permanent residents or who spend the night in the selected households the night before the survey were included. The study was restricted to women who were living with their youngest child (0–23 months of age) in order to minimize recall bias [4, 24–28]. A total of 15,106 women were included and the eligible women response rate of the surveys ranged from 94.6% in 2016 to 97.8% in 2000. The detailed methodology of the surveys has been reported elsewhere [24–27].

### Outcome variables

The outcome variables for this study were early initiation of breastfeeding and EBF, measured according to the WHO/UNICEF definitions for assessing infant and young child feeding (IYCF) [29].

- Early (timely) initiation of breastfeeding was defined as the proportion of children aged 0–23 months who were put to the breast within the first hour of birth.
- EBF was defined as the proportion of infants 0–5 months of age who were fed no other food or drink, not even water, except breast milk (including milk expressed or from a wet nurse), but allows the infant to receive Oral Rehydration Salt (ORS), drops, and syrups (vitamins, minerals and medicines). This based on the maternal 24 h recall.

### Study variables

Study variables were broadly categorized as socioeconomic, demographic, health service, and community level factors. These variables were selected based on data availability and previous studies conducted in low and middle income countries (including Ethiopia) which suggested an association between these factors and early initiation of breastfeeding and EBF [16, 22, 23, 28, 30].

Socioeconomic factors included maternal/partner education and occupation, as well as the household wealth index. Maternal/partner education was categorized as no schooling, primary education, or secondary and higher education. Maternal occupation was categorized in to three groups (mothers working in professional, technical, managerial, clerical, and services areas were categorized under “formal employment”; those who were working in agricultural and manual works were categorized under “informal employment” and non-working mothers were categorized under “No employment”) [31]. The EDHS used the principal components analysis to calculate the household wealth index based on a series of variables relating to ownership of household assets such as radio and bicycles, type of materials used for housing construction, and type of water source and sanitation facilities. Household wealth index was categorized as poor, middle or rich.

Demographic factors included maternal age (categorized as 15–24 years, 25–34 years or 35–49 years), gender of the baby (male or female), birth order (categorized as 1, 2–4 or 5 and above), family size (categorized as ≤3, 4–5, or 6 and above members), and mothers desire for pregnancy (categorized as desired the pregnancy or not desired the pregnancy).

Health service factors included frequency of antenatal visits (ANC, categorized as none, 1–3 visits, or 4 and above visits), timing of postnatal checkup (categorized as no visits, within a week or after a week), place of delivery (categorized as home or health facility), delivery assistance (categorized as a health professional, traditional birth attendants, or other untrained individuals), and mode of delivery (categorized as cesarean section or vaginal birthing).

The community level factors included a geographical region of residence and place of residence. The geographical region was recategorized into three regions (larger central, small peripherals or metropolis) based on their geopolitical features, consistent with a previous study from Ethiopia [32]. The larger central regions include Tigray, Amhara, Oromia, and Sothern Nations Nationalities and Peoples Region (SNNPRs). Small peripherals include Afar, Somali, Benishangul, and Gambella, while Metropolis include Harari, Dire Dawa, and Addis Ababa regions. Place of residence was categorized as urban or rural residence. Among the study participants, the majority (71.5%) of the mothers had no formal education, while approximately 55.0% of respondents were a housewife or unemployed [see Additional file 1].

### Statistical analysis

Preliminary analyses involved the estimation of the study factors (socioeconomic, demographic, health service and community level factors) for each year of the survey. This was followed by the calculation of the prevalence of early initiation of breastfeeding and EBF by the study factors to assess the extent to which the prevalence decreased or increased over the study period (2000–2016).

A four-staged multivariate logistic regression modelling was used to investigate the association between the study factors and early initiation of breastfeeding and EBF in each year of the survey. In the first stage, the association between socioeconomic factors and the outcome variables was examined, while adjusting for demographic, health service and community level factors as confounding variables based on previously published studies [2, 28, 30, 33, 34]. In the second stage, demographic factors were entered into the model to examine their relationship with the outcome variables, adjusting for socioeconomic, health service and community level factors. In subsequent models (stage three and four), similar analytical strategies were used in examining the association between health service and community level factors and the outcomes variables, respectively.

In this study, the data were combined in order to increase the statistical power of the study given the narrow age limit for EBF (0–5 months). In models of the combined data, a similar four-stage analytical approach was used, as well as adjustment for year of the survey to estimate the association between the study factors and breastfeeding outcomes over time. Adjusted Odds ratios (aORs) with 95% confidence intervals (CI) were calculated as the measure of association between the study factors and outcome variables. We also tested for multicollinearity in the models but no significant results were evident in the analyses. All analyses were conducted using ‘svy’ command for counts and percentages, and ‘melogit’ command for the regression analyses to adjust for sampling weights, clustering and stratification in Stata (version 14.0, Stata Corp, College Station, TX, USA) [35].

## Results

### Prevalence of early initiation of breastfeeding and EBF by the study factors

Over the study period, mothers who delivered at the health facility (67.1%) had the highest prevalence of early initiation of breastfeeding, followed by those who were from the metropolitan region (66.6%) [Table 1]. Mothers who gave birth through cesarean section had the lowest prevalence of early initiation of breastfeeding (38.8%) [Table 1]. Mothers who were in informal employment had the highest prevalence of EBF (60.2%), followed by older mothers (35–49 years, 56.6%). The lowest prevalence of EBF was in mothers who resided in the four peripheral regions (Afar, Somali, Benishangul, and Gambella) of Ethiopia (30.1% in all regions) [Table 2].

**Table 1:** Prevalence of early initiation of breastfeeding by study factors in Ethiopia, 2000–2016

Variables	2000	2005	2011	2016	2000–2016
	n (%)	n (%)	n (%)	n (%)	n (%)
Socioeconomic factors					
Maternal education					
No schooling	1678 (49.0)	2147 (69.0)	1446 (51.9)	1820 (75.8)	7092 (60.5)
Primary school	291 (50.2)	455 (65.1)	637 (53.6)	948 (76.2)	2331 (62.8)
Secondary and higher	90 (40.9)	114 (63.6)	123 (65.8)	257 (73.0)	583 (62.2)
Maternal employment					
No employment	847 (51.9)	2037 (70.3)	1179 (56.9)	1794 (76.3)	5858 (65.4)
Formal employment	168 (45.2)	178 (63.9)	361 (53.1)	397 (79.2)	1104 (60.3)
Informal employment	1044 (47.1)	501 (62.5)	648 (47.1)	833 (72.9)	3025 (54.6)
Partner education					
No schooling	1207 (46.5)	1522 (68.7)	995 (50.2)	1269 (74.5)	4993 (58.8)
Primary school	592 (54.2)	878 (68.5)	979 (56.1)	1200 (78.2)	3649 (64.5)
Secondary and higher	233 (48.9)	286 (64.8)	206 (55.1)	416 (74.6)	1141 (61.7)
Household wealth status					
Poor	643 (49.8)	1191 (70.1)	963 (50.7)	1376 (76.3)	4174 (62.3)
Middle	539 (45.3)	603 (69.0)	453 (52.1)	639 (76.0)	2235 (59.2)
Rich	876 (50.4)	022 (65.6)	789 (56.7)	1010 (74.6)	3597 (61.1)
Demographic factors					
Maternal age					
15–24 years	667 (47.5)	835 (66.4)	658 (51.8)	878 (75.0)	3038 (59.5)
25–34 years	992 (51.5)	1292 (68.5)	1090 (52.80	1574 (77.5)	4949 (63.6)
35–49 years	340 (45.0)	590 (70.6)	458 (55.2)	572 (72.1)	2019 (60.3)
Child sex					
Male	1057 (48.7)	401 (65.1)	307 (46.1)	368 (68.4)	4946 (59.6)
Female	1002 (48.9)	349 (67.4)	293 (51.6)	410 (76.5)	5059 (62.7)
Family size					
≤ 3	219 (46.5)	281 (66.9)	239 (49.4)	337 (71.7)	1076 (58.3)
4–5	706 (49.0)	885 (67.5)	788 (55.0)	1057 (76.5)	3435 (61.7)
6+	1134 (49.1)	1551 (69)	1178 (52.5)	1631 (76.0)	5494 (61.4)
Desire for pregnancy					
Desired the pregnancy	1736 (50.4)	2232 (68.2)	1998 (53.7)	2794 (76.1)	8760 (62.1)
Not desired the pregnancy	320 (41.3)	485 (68.6)	208 (47.2)	231 (71.4)	1244 (55.3)
Health service factors					
Antenatal Visit					
None	1544 (49.7)	2011 (71.0)	1207 (51.2)	1071 (78.1)	5833 (60.3)
1–3 visits	328 (47.6)	423 (63.9)	569 (53.0)	946 (74.2)	1266 (61.3)
4+ visits	174 (44.2)	271 (57.3)	427 (58.9)	1004 (74.6)	1876 (63.9)
Mode of delivery					
Vaginal birthing	2049 (48.9)	2699 (68.4)	2178 (53.3)	2984 (76.7)	9910 (61.5)
Cesarean section	8 (33.3)	18 (48.6)	28 (35.6)	40 (38.9)	94 (38.8)
Place of birth					
Health facility	79 (38.0)	141 (59.5)	260 (55.4)	1117 (76.1)	1596 (67.1)
Home	1980 (49.3)	2574 (68.8)	1946 (52.7)	1907 (75.4)	8407 (60.1)
Delivery assistance					
Health professional	175 (45.7)	276 (64.7)	266 (54.8)	1130 (75.6)	1846 (66.2)
Traditional birth attendants	390 (46.8)	333 (64.5)	127 (50.9)	1029 (76.3)	1879 (63.7)
Other untrained individuals	1369 (49.5)	1930 (68.6)	1734 (52.9)	401 (72.3)	5434 (57.7)
Community level factors					
Place of residence					
Urban	180 (44.9)	184 (63.8)	325 (59.3)	368 (76.8)	1056 (61.6)
Rural	1879 (49.2)	2533 (68.6)	1881 (52.0)	2657 (75.5)	8949 (61.1)
Region of residence					
Large central regions	1953 (48.8)	2463 (67.1)	2033 (52.9)	2742 (75.8)	9191 (60.7)
Small peripheral regions	60 (45.8)	199 (86.3)	100 (46.7)	187 (74.2)	546 (66.0)
Metropolis	46 (51.6)	555 (78.3)	73 (66.9)	96 (74.7)	269 (66.6)

n (%): weighted count and proportion for each outcome variable by study factors

**Table 2:** Prevalence of exclusive breastfeeding by study factors in Ethiopia, 2000–2016

Variables	2000	2005	2011	2016	2000–2016
	n (%)	n (%)	n (%)	n (%)	n (%)
Socioeconomic factors					
Maternal education					
No schooling	489 (56.2)	454 (51)	434 (53.7)	392 (60.1)	1769 (54.9)
Primary school	71 (51.5)	96 (46.5)	184 (49.1)	101 (60.7)	552 (52.6)
Secondary and higher	21 (36.0)	10 (21.6)	30 (48.0)	64 (59.9)	125 (44.7)
Maternal employment					
No employment	207 (49.2)	451 (49.0)	329 (49.5)	395 (56.8)	1382 (51.2)
Formal employment	44 (48.6)	15 (27.4)	79 (44.1)	82 (66.3)	221 (49.1)
Informal employment	330 (59.5)	93 (56.3)	235 (59.7)	180 (65.0)	838 (60.2)
Partner education					
No schooling	352 (56.6)	322 (49.9)	313 (54.5)	316 (62.4)	1302 (55.5)
Primary school	157 (53.1)	180 (51.1)	275 (52.4)	226 (58.4)	837 (53.8)
Secondary and higher	62 (48.0)	52 (41.0)	43 (34.1)	96 (59.3)	254 (46.5)
Household wealth status					
Poor	204 (57.4)	202 (44.3)	292 (51.9)	321 (61.4)	1019 (53.7)
Middle	143 (49.4)	151 (58.9)	168 (59.4)	115 (56.7)	576 (56.0)
Rich	235 (55.6)	207 (49.1)	189 (46.9)	221 (59.7)	852 (52.4)
Demographic factors					
Maternal age					
15–24 years	225 (54.9)	209 (47.8)	204 (47.7)	220 (59.6)	857 (52.2)
25–34 years	257 (53.4)	237 (46.7)	313 (54.8)	320 (60.1)	1126 (53.8)
35–49 years	100 (56.7)	114 (57.6)	132 (53.0)	118 (60.1)	464 (56.6)
Child sex					
Male	284 (51.5)	303 (49.0)	323 (48.2)	316 (58.2)	1226 (51.5)
Female	298 (57.8)	256 (48.9)	326 (56.4)	341 (61.6)	1221 (56.3)
Birth order					
One	88 (48.4)	98 (45.9)	108 (46.8)	135 (51.0)	429 (48.2)
2–4	254 (53.5	250 (47.6)	289 (53.01)	293 (65.5)	1087 (54.5)
5+	239 (58.4)	212 (52.5)	252 (53.4)	229 (60.0)	931 (55.8)
Family size					
≤ 3	46 (41.0)	54 (46.5)	52 (41.2)	50 (46.3)	202 (43.6)
4–5	179 (52.4)	184 (47.8)	211 (52.5)	235 (65.6)	808 (54.4)
6+	367 (58.2)	322 (50.2)	386 (53.6)	372 (59)	1437 (55.2)
Desire for pregnancy					
Desired the pregnancy	457 (53.3)	439 (46.9)	569 (51.8)	625 (62.0)	2090 (53.6)
Not desired the pregnancy	124 (59.4)	121 (58.4)	80 (53.4)	32 (36.5)	357 (54.7)
Listening radio					
No	442 (55.4)	371 (51.2)	311 (52.5)	492 (61.3)	1617 (55.4)
Yes	139 (51.7)	187 (45.1)	338 (51.5)	166 (56.1)	830 (50.8)
Health service factors					
Antenatal Visit					
None	436 (56.6)	425 (52.2)	363 (53.4)	210 (53.9)	1434 (54.0)
1–3 visits	106 (51.0)	96 (46.5)	195 (53.3)	232 (62.0)	628 (54.5)
4+ visits	39 (44.2)	38 (32.0)	91 (45.0)	213 (64.3)	381 (51.4)
Place of birth					
Health facility	23 (37.2)	22 (34.4)	67 (48.0)	254 (62.2)	366 (54.4)
Home	559 (55.6)	537 (49.9)	582 (52.5)	404 (58.6)	2081 (53.6)
Delivery assistance					
Health professional	44 (42.3)	53 (41.0)	68 (47.1)	257 (62.1)	422 (53.3)
Traditional birth attendants	109 (51.5)	81 (54.8)	32 (41.0)	225 (58.7)	447 (54.5)
Other untrained individuals	390 (57.2)	411 (50.4)	542 (53.5)	67 (49.8)	1410 (53.3)
Timing of postnatal checkup					
None	556 (54.6)	536 (49.5)	633 (52.4)	622 (60.4)	2348 (54.1)
Within a week	21 (54.7)	14 (31.8)	3 (66.8)	21 (60.1)	58 (48.5)
After a week	5 (49.1)	10 (57.8)	13 (36.9)	14 (44.4)	42 (44.5)
Community level factors					
Place of residence					
Urban	47 (45.6)	33 (42.5)	67 (43.8)	78 (60.9)	225 (48.8)
Rural	535 (55.4)	527 (49.5)	582 (53.2)	579 (69.8)	2222 (54.3)
Region of residence					
Large central regions	56 (56.6)	540 (51.5)	620 (54.2)	604 (60.8)	2330 (55.7)
Small peripheral regions	10 (25.7)	15 (19.8)	19 (24.7)	35 (48.8)	78 (30.1)
Metropolis	5 (17.9)	5 (26.1)	11 (36.9)	18 (58.1)	39 (36.3)

n (%): weighted count and proportion for each outcome variable by study factors

In 2016, the prevalence of early initiation of breastfeeding was highest in children whose father’s had primary education (78.2%), while the lowest prevalence was among mothers who gave birth through cesarean section (38.9%) (Table 1). In the same year, mothers who were in a formal employment (66.3%) and those who did not desire a pregnancy (36.5%) had the highest and the lowest EBF prevalence, respectively (Table 2).

### Trends in early initiation of breastfeeding and EBF in Ethiopia, 2000–2016

**Fig. 1:**
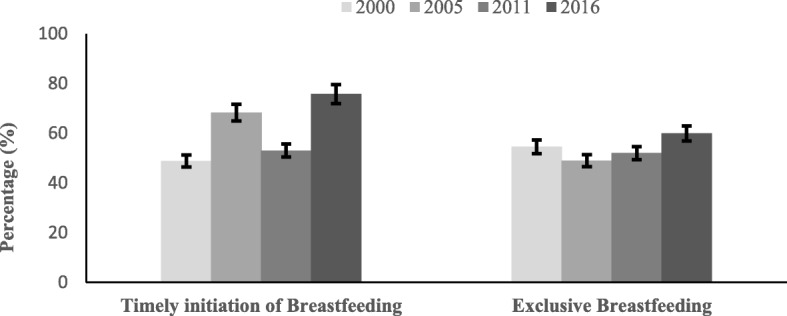
Trends in early initiation of breastfeeding and exclusive breastfeeding in Ethiopia, 2000–2016. Error bars indicate 95% confidence intervals

The prevalence of early initiation of breastfeeding increased from 48.8% (95% Confidence Interval [CI] 45.4, 52.2%) in 2000 to 75.7% (95% CI 73.0, 78.0%) in 2016. However, the progress of EBF was not significant in these years which was 54.5% (95% CI 49.9, 59.0%) in 2000 and 59.9% (95% CI 55, 64.5%) in 2016 (Fig. 1).

### Determinants of early initiation of breastfeeding

Between 2000 and 2016, mothers who were informally employed were less likely to initiate breastfeeding within the first hour of birth compared to those who were unemployed (Adjusted Odds Ratio [aOR] 0.75; 95% CI 0.68, 0.83). Children whose fathers attended primary education had increased odds of early initiation of breastfeeding compared to those whose father had no education (aOR 1.14; 95% CI 1.03, 1.26). Mothers who did not desire the current pregnancy (aOR 0.81; 95% CI 0.71, 0.91) and those who gave birth through a cesarean section (aOR 0.22; 95% CI 0.17, 0.30) had lower odds of early initiation of breastfeeding than their counterparts. The likelihood of early initiation of breastfeeding among mothers who gave birth at a health facility was higher as compared to those who gave birth at home (aOR 1.35; 95% CI 1.05, 1.75). Mothers who resided in the metropolis region of Ethiopia were more likely to initiate breastfeeding within the first hour of birth as compared to those who were from larger central regions (aOR 1.95; 95% CI 1.65, 2.32) (Table 3).

**Table 3:** Determinants of early initiation of breastfeeding in Ethiopia, 2000–2016

Variables	2000	2005	2011	2016	2000–2016	*p* for trend
	^a^aOR (95% CI)	^a^aOR (95% CI)	^a^aOR (95% CI)	^a^aOR (95% CI)	^a^aOR (95% CI)	
Socioeconomic factors						
Maternal education						
No schooling	1.00	1.00	1.00	1.00	1.00	*P* < 0.001
Primary school	0.88 (0.67, 1.15)	0.96 (0.73, 1.25)	1.00 (0.81, 1.24)	1.15 (0.90, 1.47)	0.99 (0.89, 1.11)	*P* < 0.001
Secondary and higher	0.83 (0.50, 1.38)	1.24 (0.72, 2.13)	1.27 (0.81, 2.00)	1.30 (0.87, 1.93)	1.05 (0.85, 1.30)	*P* < 0.001
Partner education						
No schooling	1.00	1.00	1.00	1.00	1.00	*P* < 0.001
Primary school	1.24 (0.99, 1.56)	0.96 (0.76, 1.20)	1.06 (0.89, 1.27)	1.05 (0.81, 1.34)	1.14 (1.03, 1.26)	*P* < 0.001
Secondary and higher	1.04 (0.73, 1.46)	0.92 (0.64, 1.33)	0.89 (0.65, 1.24)	0.98 (0.71, 1.35)	1.01 (0.86, 1.19)	*P* < 0.001
Maternal employment						
No employment	1.00	1.00	1.00	1.00	1.00	*P* < 0.001
Formal employment	1.00 (0.72, 1.39)	0.72 (0.52, 0.98)	0.82 (0.65, 1.03)	1.54 (1.07, 2.21)	0.94 (0.82, 1.07)	*P* < 0.001
Informal employment	0.79 (0.64, 0.98)	0.71 (0.54, 0.93)	0.74 (0.61, 0.90)	1.00 (0.79, 1.28)	0.75 (0.68, 0.83)	*P* < 0.001
Household wealth index						
Poor	1.00	1.00	1.00	1.00	1.00	*P* < 0.001
Middle	1.00 (0.86, 1.18)	1.02 (0.80, 1.29)	1.04 (0.85, 1.27)	1.19 (0.87, 1.62)	1.08 (0.97, 1.21)	*P* < 0.001
Rich	0.99 (0.80, 1.23)	0.97 (0.74, 1.28)	1.22 (0.98, 1.53)	1.20 (0.87, 1.65)	1.13 (1.01, 1.26)	*P* < 0.001
Demographic factors						
Maternal age						
15–24 years	1.00	1.00	1.00	1.00	1.00	*P* < 0.001
25–34 years	1.14 (0.94, 1.38)	1.05 (0.85, 1.29)	1.16 (0.96, 1.41)	1.20 (0.94, 1.52)	1.12 (1.02, 1.23)	*P* < 0.001
35–49 years	1.08 (0.84, 1.38)	0.99 (0.75, 1.31)	1.29 (0.99, 1.68)	1.26 (0.91, 1.74)	1.13 (0.99,1.27)	*P* < 0.001
Sex of the baby						
Male	1.00	1.00	1.00	1.00	1.00	*P* < 0.001
Female	1.00 (0.86, 1.18)	0.99 (0.84, 1.19)	1.14 (0.98, 1.32)	1.21 (1.01, 1.45)	1.08 (1.00, 1.16)	*P* < 0.001
Desire for pregnancy						
Desired the pregnancy	1.00	1.00	1.00	1.00	1.00	*P* < 0.001
Not desired the pregnancy	0.83 (0.67, 1.04)	0.82 (0.0.64, 1.06)	0.69 (0.52, 0.91)	1.10 (0.66, 1.84)	0.81 (0.71, 0.91)	*P* < 0.001
Health service factors						
Antenatal care visit						
None	1.00	1.00	1.00	1.00	1.00	*P* = 0.001
1–3 visits	0.71 (0.56, 0.91)	0.65 (0.51, 0.84)	0.98 (0.81, 1.18)	0.82 (0.63, 1.06)	0.77 (0.70, 0.86)	*P* < 0.001
4+ visits	0.76 (0.54, 1.05)	0.51 (0.37, 0.69)	0.94 (0.73, 1.21)	0.75 (0.57, 0.98)	0.74 (0.65, 0.85)	*P* < 0.001
Place of birth						
Home	1.00	1.00	1.00	1.00	1.00	*P* = 0.695
Health facility	0.97 (0.57, 1.66)	0.79 (0.46, 1.33)	1.16 (0.53, 2.56)	2.03 (1.04, 4.00)	1.35 (1.05, 1.75)	*P* < 0.001
Mode of delivery						
Vaginal birthing	1.00	1.00	1.00	1.00	1.00	*P* < 0.001
Cesarean section	0.42 (0.20, 0.92)	0.30 (0.15, 0.58)	0.19 (0.11, 0.35)	0.10 (0.05, 0.16)	0.22 (0.17, 0.30)	*P* < 0.001
Community factors						
Region of residence						
Large central regions	1.00	1.00	1.00	1.00	1.00	*P* < 0.001
Small peripheral regions	1.24 (0.93, 1.66)	2.39 (1.72, 3.32)	1.03 (0.81, 1.30)	0.82 (0.63, 1.07)	1.13 (0.99, 1.29)	*P* = 0.006
Metropolis	1.81 (1.31, 2.50)	3.03 (1.93, 4.75)	2.13 (1.60, 2.84)	2.65 (1.79, 3.93)	1.95 (1.65, 2.32)	*P* < 0.001

^a^aORs of socioeconomic factors were adjusted for demographic, health service and community level factors; aORs of demographic factors were adjusted for socioeconomic, health service and community level factors; aORs of health service factors were adjusted for socioeconomic, demographic, and community level factors; aORs of community level factors were adjusted for socioeconomic, demographic, and health service

In the 2016 data, formally employed mothers (aOR 1.54; 95% CI 1.07, 2.21) and children who were born at a health facility (aOR 2.03; 95% CI 1.04, 4.00) were significantly associated with early initiation of breastfeeding. In the same year, cesarean birthing (aOR 0.10; 95% CI 0.05, 0.16) and frequent antenatal care visits (aOR 0.75; 95% CI 0.57, 0.98) were associated with delayed initiation of breastfeeding (Table 3).

### Determinants of EBF

In the combined data, informally employed mothers were more likely to exclusively breastfed their babies until 6 months compared to those who had no employment (aOR 1.37; 95% CI 1.15, 1.63). Mothers who were from middle level households were more likely to engage in EBF compared to those who were from poor households (aOR 1.21; 95% CI 1.04, 1.53). The likelihood of EBF was also higher among children who were living with 4–5 (aOR 1.36; 95% CI 1.04, 1.79), and six and above (aOR 1.46; 95% CI 1.10, 1.93) family members than two family members. The odds of EBF was lower among mothers who are from metropolis (aOR 0.46; 95% CI 0.36, 0.58), and small peripheral regions (aOR 0.37; 95% CI 0.31, 0.45) compared to those who were from larger central regions (Table 4).

**Table 4:** Determinants of exclusive breastfeeding in Ethiopia, 2000–2016

Variables	2000	2005	2011	2016	2000–2016	*p* for trend
	^a^aOR (95% CI)	^a^aOR (95% CI)	^a^aOR (95% CI)	^a^aOR (95% CI)	^a^aOR (95% CI)	
Socioeconomic factors						
Maternal education						
No schooling	1.00	1.00	1.00	1.00	1.00	*P* < 0.001
Primary school	0.89 (0.49, 1.59)	0.89 (0.53, 1.48)	0.90 (0.62, 1.33)	1.39 (0.88, 2.19)	0.98 (0.81, 1.20)	*P* = 0.003
Secondary and higher	0.61 (0.19, 1.89)	0.69 (0.25, 1.95)	1.47 (0.59, 3.64)	1.48 (0.74, 2.97)	0.99 (0.68, 1.44)	*P* = 0.001
Maternal employment						
No employment	1.00	1.00	1.00	1.00	1.00	*P* < 0.001
Formal employment	1.56 (0.76, 3.20)	0.53 (0.25, 1.11)	1.03 (0.63, 1.69)	0.99 (0.58, 1.67)	0.94 (0.72, 1.21)	*P* = 0.004
Informal employment	1.71 (1.17, 2.49)	1.06 (0.66, 1.71)	1.27 (0.89, 1.82)	1.14 (0.74, 1.75)	1.37 (1.15, 1.63)	*P* = 0.011
Household wealth index						
Poor	1.00	1.00	1.00	1.00	1.00	*P* = 0.001
Middle	0.72 (0.47, 1.09)	1.93 (1.22, 3.06)	1.43 (0.97, 2.12)	1.03 (0.61, 1.72)	1.21 (1.04, 1.53)	*P* < 0.001
Rich	1.00 (0.67, 1.48)	1.54 (0.99, 2.38)	1.02 (0.65, 1.59)	0.85 (0.51, 1.42)	1.20 (0.99, 1.45)	*P* < 0.001
Demographic factors						
Maternal age						
15–24 years	1.00	1.00	1.00	1.00	1.00	*P* < 0.001
25–34 years	0.81 (0.52, 1.26)	1.03 (0.66, 1.60)	0.86 (0.59, 1.25)	1.04 (0.68, 1.59)	0.87 (0.72, 1.05)	*P* < 0.001
35–49 years	0.97 (0.51, 1.82)	1.13 (0.60, 2.11)	0.80 (0.47, 1.37)	1.22 (0.64, 2.33)	0.91 (0.69, 1.20)	*P* = 0.653
Birth order						
One	1.00	1.00	1.00	1.00	1.00	*P* < 0.001
2–4	0.70 (0.42, 1.27)	0.83 (0.49, 1.41)	0.99 (0.62, 1.57)	1.34 (0.84, 2.13)	0.95 (0.76, 1.20)	*P* < 0.001
5+	0.55 (0.29, 1.04)	0.96 (0.50, 1.87)	0.71 (0.40, 1.26)	1.09 (0.56, 2.10)	0.85 (0.63, 1.15)	*P* = 0.035
Family size						
≤ 3	1.00	1.00	1.00	1.00	1.00	*P* = 0.001
4–5	1.89 (1.00, 3.55)	1.32 (0.72, 2.41)	1.86 (1.09, 3.17)	1.01 (0.55, 1.85)	1.36 (1.04, 1.79)	*P* < 0.001
6+	2.61 (1.38, 4.94)	1.19 (0.63, 2.25)	2.09 (1.18, 3.67)	1.01 (0.54, 1.89)	1.46 (1.10, 1.93)	*P* < 0.001
Listening radio						
No	1.00	1.00	1.00	1.00	1.00	*P* < 0.001
Yes	1.00 (0.67, 1.52)	0.81 (0.56, 1.17)	1.10 (0.80, 1.52)	0.72 (0.47, 1.10)	0.90 (0.76, 1.06)	*P* < 0.001
Health service factors						
Antenatal visit						
None	1.00	1.00	1.00	1.00	1.00	*P* = 0.018
1–3 visits	0.69 (0.45, 1.08)	0.97 (0.66, 1.43)	0.93 (0.64, 1.35)	1.63 (1.06, 2.49)	0.97 (0.80, 1.17)	*P* < 0.001
4+ visits	0.86 (0.44, 1.71)	0.71 (0.39, 1.28)	0.77 (0.45, 1.30)	2.26 (1.46, 3.50)	1.01 (0.79, 1.30)	*P* < 0.001
Timing of postnatal checkup						
None	1.00	1.00	1.00	1.00	1.00	*P* = 0.145
Within a week	0.67 (0.33, 1.33)	1.11 (0.43, 2.85)	1.25 (0.29, 5.31)	0.92 (0.40, 2.10)	0.84 (0.57, 1.22)	–
After a week	0.76 (0.23, 2.47)	0.87 (0.30, 2.49)	0.81 (0.23, 2.84)	0.54 (0.23, 1.29)	0.69 (0.41, 1.16)	*P* < 0.001
Community level factors						
Place of residence						
Urban	1.00	1.00	1.00	1.00	1.00	*P* < 0.001
Rural	0.77 (0.40, 1.49)	0.81 (0.38, 1.70)	1.01 (0.51, 2.00)	1.32 (0.76, 2.31)	1.08 (0.80, 1.45)	*P* < 0.001
Region of residence						
Large central regions	1.00	1.00	1.00	1.00	1.00	*P* < 0.001
Small peripheral regions	0.24 (0.16, 0.38)	0.30 (0.19, 0.49)	0.27 (0.18, 0.40)	0.66 (0.44, 0.90)	0.37 (0.31, 0.45)	*P* = 0.015
Metropolis	0.16 (0.09, 0.30)	0.37 (0.18, 0.76)	0.56 (0.33, 0.95)	0.83 (0.54, 1.28)	0.46 (0.36, 0.58)	*P* < 0.001

^a^aORs of socioeconomic factors were adjusted for demographic, health service and community level factors; aORs of demographic factors were adjusted for socioeconomic, health service and community level factors; aORs of health service factors were adjusted for socioeconomic, demographic, and community level factors; aORs of community level factors were adjusted for socioeconomic, demographic, and health service

In the 2016 data, the odds of EBF was higher among mothers who reported 1–3 (aOR 1.63; 95% CI 1.06, 2.49) and four or more ANC visits (aOR 2.26; 95% CI 1.46, 3.50) compared to mothers who had no ANC visits. However, mothers who resided in small peripheral regions were less likely to practice EBF compared to those who resided in the larger central regions (aOR 0.66; 95% CI 0.44, 0.90) (Table 4).

## Discussion

The present study indicated that early initiation of breastfeeding increased from 48.8% in 2000 to 75.5% in 2016, potentially reflecting the impacts of the national infant and young child feeding strategy in Ethiopia [36]. The study also found that EBF improved from 54.5% in 2000 to 59.9% in 2016, but this was not statistically significant. Between 2000 and 2016, maternal informal employment, frequent ANC visits and cesarean delivery were associated with delayed initiation of breastfeeding, while delivery at a health facility and residing in the metropolis region were associated with a higher prevalence of early initiation of breastfeeding. In the combined data, informally employed mothers and those with four or more members in the family were more likely to exclusively breastfed their babies. In the 2016 data, maternal formal employment, giving birth at a health facility, and residing in the metropolis region were associated with early initiation of breastfeeding. In similar data, frequent ANC visit was associated with EBF, while those resided in metropolis region were less likely to exclusively breastfed.

During the MDG period (2000–2015), which is almost similar to the study period, maternal employment and region of residence were the common factors associated with both early initiation of breastfeeding and EBF in Ethiopia. The odds of engaging in early initiation of breastfeeding was lower among mothers who were informally employed; however, EBF prevalence was higher in this group. The relationship between informal employment and early initiation of breastfeeding could probably be explained by poor awareness and unfavorable sociocultural practices (like prelacteal feeding) that is common among mothers employed in agricultural or manual works [37]. Globally, there is variation in the literature about the influence of maternal employment on the early initiation of breastfeeding. For example, employed mothers from Nigeria [28], Namibia [38], and Pakistan [39] were more likely to delay initiation of breastfeeding compared to mothers not in employment. Evidence from India, nonetheless, suggested that employed mothers were less likely to delay initiation of breastfeeding [40]. The relationship between maternal informal employment and EBF might be explained by the flexible working hours that mothers in the informal sectors have as it allows them to breastfeed their babies on demand [41]. However, subnational studies conducted in Ethiopia indicated that maternal employment was associated with lower odds of exclusive breastfeeding EBF [42, 43]. Evidence from the Middle East [31, 44] was consistent with our finding, where mothers informal employment or working outside their homes were less likely to engage in EBF compared with those in informal employment.

Mothers from the three metropolitan regions (Addis Ababa, Dire Dawa, and Harar) had higher odds of engaging in early initiation of breastfeeding, but had a lower likelihood of exclusive breastfeeding. This finding was supported by evidence from a systematic review conducted in Ethiopia, where mothers residing in urban areas had the highest proportion of early initiation of breastfeeding, but also the lowest prevalence of EBF compared to rural areas [16]. A possible explanation for the higher prevalence of early initiation of breastfeeding in urban mothers may be that mothers residing in urban areas may have higher educational levels, with potential improved access to breastfeeding information [38]. Nevertheless, there is a disparity in the literature on the influence of place of residence on early initiation of breastfeeding worldwide. For instance, mothers who live in rural Nigeria [30], Sri Lanka [45] and India [40] were more likely to delay breastfeeding initiation compared to those who live in urban Nigeria, Sri Lanka and India. However, mothers residing in rural Bangladesh were more likely to initiate breastfeeding post-birth compared to their counterparts in urban Bangladesh [46]. A possible explanation for the decreased likelihood of EBF might be that metropolitan regions probably have a higher number of formally employed women [41].

In many LMIC countries (including Ethiopia), early initiation of breastfeeding is influenced by father’s education and household wealth [23, 28, 47]. The present study suggested that father’s education and household wealth were associated with initiation of breastfeeding within the first hour of birth, consistent with studies from Nigeria, Nepal, and Australia [30, 48, 49]. Educated fathers are more likely to be formally employed, with subsequent impact on the household wealth and productivity [50, 51]. This can also translate to increase breastfeeding awareness that might influence the mother to engage in optimal breastfeeding because partners plays a role an important role in a mother’s decision to initiate and continue optimal breastfeeding [52, 53]. The relationship between household wealth and early initiation of breastfeeding was demonstrated by a recent evidence from Nigeria, which suggested that 25.0% of non-EBF were attributable to lower household wealth [54]. In Ethiopia, health practitioners and policy-decision makers would do well to involve partners in breastfeeding promotion and support programs to maximize gains of breastfeeding interventions. In addition, improving human capital resources, particularly among women, in line with the current SDG-1.1 of eradicate extreme poverty and (SDG-4.3) of ensuring access to education for all women would be central to increasing breastfeeding outcomes in Ethiopia [13].

Research from nine sub-Saharan African countries indicated that frequent ANC visits was associated with early initiation of breastfeeding [55], possibly reflecting the impact of ANC services, including promotion of breastfeeding message by health professionals [55]. However, our study suggested that mothers who had frequent (≥ 4) ANC visits were more likely to delay breastfeeding initiation within the first hour of birth. This finding is consistent with research from Namibia, where frequent ANC visits were associated with delayed breastfeeding initiation post-birth [38]. A plausible reason for this finding may be that health practitioners have limited knowledge about optimal infant feeding [56] or an absolute lack of accredited Baby-Friendly Hospital Initiative (BFHI) facilities in Ethiopia [57]. This indicates that while many mothers may have received routine ANC information, they may have had limited opportunities to obtain appropriate breastfeeding information. Additionally, the association between frequent ANC visits and delayed initiation of breastfeeding may also be due to problematic pregnancies or ill health experienced by mothers, which may require frequent health service visits. Nonetheless, initiating and implementing the BFHI strategy would be crucial in assisting efforts to implement breastfeeding practices that protect, promote and support appropriate breastfeeding in Ethiopia.

Our study indicated that health facility birthing was associated with early initiation of breastfeeding, and this was in line with a systematic review from Ethiopia [58] and studies from Nigeria [28], Namibia [38], and Uganda [59]. The practice of EBF in the early postnatal period is affected by many factors, even when there is delay in breastfeeding initiation. These factors include cesarean birthing [20, 30, 52], cultural practices [37, 60], and influence of grandmothers [61]. Importantly, a woman’s engagement with the health facility in the perinatal period remains a major determining factor in ensuring optimal infant feeding behavior. This health facility engagement does not only provide relevant and appropriate information to the woman but also gives the woman confidence and information to challenge perceived cultural practices, myths and belief system held towards breastfeeding in the community [62]. In Ethiopia, interventions to increase the pace of progress for EBF should involve the health facilities, including training of health practitioners, accrediting health facilities for BFHI, and evaluating the quality of health services provided.

Young mothers, female children, and mothers who desired the pregnancy were associated with higher odds of early initiation of breastfeeding. Mothers who were in the age group of 25–34 years had significantly higher odds of early initiation of breastfeeding which might be explained that they are in the peak of reproductive age group to start a family. A study conducted in Bangladesh showed that teenage motherhood was associated with delayed initiation of breastfeeding In the current study, female infants had a higher odds of early initiation of breastfeeding compared with male children which is similar to a study conducted in regional Ethiopia [63]. The relationship between a woman’s desire for pregnancy and early initiation of breastfeeding was supported by a similar study conducted in Turkey, which reported that unwanted pregnancy decreases the odds of early initiation of breastfeeding [64]. This finding could be explained by the compromised attitude of the mother towards the baby, which may affect her intention to initiate breastfeeding, as well as her decision for optimal baby care in general [64]. Additionally, well conducted qualitative studies may be needed to understand the reasons for why EBF prevalence has not substantially increased in Ethiopia to inform focused interventions.

### Study limitations and strengths

The following limitations should be considered while interpreting the study results. Firstly, the cross-sectional nature of the data does not allow the establishment of causality. However, evidence from this study is potentially relevant to breastfeeding efforts in Ethiopia. Secondly, the survey used a 24-h recall method for measuring EBF which might be a source of recall or measurement bias. Nonetheless, an attempt was made to minimize the recall bias by restricting the analyses to youngest child, living with the mother, in line with past studies and EDHS [4, 24–28]. Finally, the study did not examine psychological and cultural factors, information that may be useful in investigating key associated factors with early initiation of breastfeeding and EBF could have been used to determine community level influential factors [52]. Despite these limitations, the use of nationally representative data can be considered a strength of this study. The survey was also applied a standard questionnaire which might minimize the effect of measurement bias.

## Conclusion

The present study showed that early initiation of breastfeeding improved in Ethiopia during the MDG era but it is still below the national target; while the progress in EBF remained slow. Improving breastfeeding practices of Ethiopian mothers and ensuring that national breastfeeding targets are met would require targeted efforts at the individual, health facility and community levels. It is also important that national and subnational policy-makers and nutrition expert in Ethiopia consider successful intervention strategies from evidence-based studies when designing breastfeeding programs.

## Supplementary information

Additional file 1. Characteristics of the study participants in Ethiopia, 2000–2016. (PDF 325 kb)

## Data Availability

The analysis was based on the datasets collected Ethiopian Demographic Health Survey. Information on the data and content can be accessed at https://dhsprogram.com/data/available-datasets.cfm

## Abbreviations

ANC: Antenatal Care
aOR: Adjusted Odds Ratio
BFHI: Baby-Friendly Hospital Initiative
CI: Confidence Interval
CSA: Central Statistics Agency
DHS: Demographic and Health Survey
EA: Enumeration Areas
EBF: Exclusive Breastfeeding
EDHS: Ethiopian Demographic and Health Survey
HSTP: Health Sector Transformation Plan
ICF: Inner City Fund
IYCF: Infant and Young Child Feeding
MDG: Millennium Development Goal
NRERC: National Research Ethics Review Committee
SDG: Sustainable Development Goals
SNNP: Southern Nations Nationalities and Peoples
UNICEF: United Nation International Children Education Fund
USAID: United States Agency for International Development
WHO: World Health Organization
