# Supplementary material for: Trends and determinants of early initiation of breastfeeding and exclusive breastfeeding in Ethiopia from 2000 to 2016
Source: Int Breastfeed J. 2019 Sep 11;14:40. doi: 10.1186/s13006-019-0234-9 (PMC6740001; doi:10.1186/s13006-019-0234-9)
Supplement: Supplementary file 1 — Characteristics of the study participants in Ethiopia, 2000–2016. (PDF 325 kb) [file 13006_2019_234_MOESM1_ESM.pdf]

Additional file 1

Characteristics of the study participants in Ethiopia, 2000–2016

Variables	2000 (N=3680)	2005 (N= 3528)	2011 (N= 4037)	2016 (N= 3861)	2000–2016 (N=15,106)
	**n (%)**	**n (%)**	**n (%)**	**n (%)**	**n (%)**
**Socioeconomic factors**					
Maternal education					
No schooling	3434 (81.1)	3120 (77.9)	2802 (66.8)	2460 (60.3)	11846 (71.5)
Primary school	582 (13.7)	705 (17.6)	1204 (28.7)	1262 (30.9)	3753 (22.7)
Secondary and higher	220 (5.2)	183 (4.5)	191 (4.5)	360 (8.8)	954 (5.8)
Maternal employment					
No employment	1640 (38.7)	2915 (72.8)	2096 (50.4)	2416 (59.2)	9066 (55.0)
Formal employment	373 (8.8)	284 (7.1)	686 (16.5)	508 (12.4)	1851 (11.2)
Informal employment	2222 (52.5)	804 (20.1)	1379 (33.1)	1160 (28.4)	5565 (33.8)
Partner education					
No schooling	2599 (62.2)	2230 (56.2)	1989 (48.1)	1744 (45.2)	8562 (53.1)
Primary school	1098 (26.3)	1289 (32.5)	1765 (42.7)	1548 (40.1)	5700 (35.3)
Secondary and higher	480 (11.5)	449 (11.3)	380 (9.2)	569 (14.7)	1878 (11.6)
Household wealth status					
Poor	129 5(30.6)	1711 (42.7)	1913 (45.6)	1846 (45.2)	6765 (40.9)
middle	1197 (28.3)	879 (21.9)	878 (20.9)	859 (21.0)	3812 (12.1)
Rich	1743 (41.2)	1417 (35.4)	1407 (33.5)	1378 (33.8)	5946 (36.0)
**Demographic factors**					
Maternal age					
15-24 years	1408 (33.3)	1267 (31.6)	1285 (30.6)	1207 ((29.6)	5167 (31.3)
25-34 years	1936 (45.7)	1900 (47.4)	2077 (49.5)	2067 (50.6)	7980 (48.3)
35-49 years	891 (21)	840 (21.0)	835 (1.9)	809 (19.8)	3375 (20.4)
Child sex					
Male	2181 (51.5)	2074 (51.8)	2166 (51.6)	1945 (47.6)	8366 (50.6)
Female	2054 (48.5)	1933 (48.2)	2031 (48.4)	2138 (52.4)	8157 (49.4)
Birth order					
One	721 (17.0)	696 (17.4)	736 (17.5)	845 (20.7)	2998 (18.1)
2-4	1840 (43.4)	1753 (43.7)	1895 (45.2)	1740 (42.6)	7228 (43.8)
5+	1675 (39.6)	1558 (38.9)	1566 (37.3)	1498 (36.7)	6297 (38.1)
Family size					
≤ 3	471 (11.1)	426 (10.6)	485 (11.6)	484 (11.8)	1866 (11.3)
4-5	1445 (34.1)	1321 (33.0)	1447 (34.5)	1411 (34.6)	5624 (34.0)
6+	2319 (54.8)	2260 (56.4)	2265 (54.9)	2188 (53.6)	9032 (54.7)
Desire for pregnancy					
Desired the pregnancy	3456 (81.6)	3293 (82.2)	3755 (89.5)	3740 (91.6)	14243 (86.2)
Not desired the pregnancy	778 (18.4)	714 (17.8)	442 (10.5)	343 (8.4)	2277 (13.8)
**Health service factors**					
Antenatal Visit					
None	3122 (74.2)	2845 (71.3)	2369 (56.6)	1412 (34.8)	9748 (59.3)
1-3	691 (16.4)	664 (16.6)	1085 (25.9)	1288 (31.7)	3727 (22.6)
4+	396 (9.4)	479 (12)	735 (17.6)	1362 (33.5)	2972 (18.1)
Mode of delivery					
Vaginal birthing	4205 (99.5)	3968 (99)	4115 (98.1)	3978 (97.4)	16266 (98.5)
Caesarean section	23 (0.5)	39 (1.0)	82 (1.9)	105 (2.6)	250 (1.5)
Place of birth					
Home	4028 (95.1)	3763 (93.9)	3721 (88.7)	2593 (63.5)	14104 (85.4)
Health facility	208 (4.9)	242 (6.1)	476 (11.4)	1490 (36.5)	2416 (14.6)
Delivery assistance					
Health professional	3397 (24.1)	432 (11.4)	491 (12.1)	1521 (43.9)	2829 (18.5)
Traditional birth attendant	3103 (22.0)	522 (13.8)	253 (6.2)	1387 (40.0)	3002 (19.6)
Others untrained	7621 (54.0)	2829 (74.8)	3304 (81.6)	560 (16.1)	9460 (61.9)
Timing of postnatal checkup					
None	4006 (94.6)	3786 (94.5)	4065 (96.9)	3776 (92.3)	15632 (94.6)
Within a week	179 (4.2)	176 (4.4)	42 (1.0)	154 (3.8)	551 (3.3)
After a week	50 (1.2)	46 (1.1)	90 (2.1)	153 (3.7)	338 (2.10)
**Community-level factors**					
Place of residence					

Urban	405 (9.6)	296 (7.4)	558 (13.3)	492 (12.0)	1750 (10.6)
Rural	3831 (90.4)	3711 (92.6)	3639 (86.7)	3591 (88.0)	14773 (89.4)
Region of residence					
Large central regions	4011 (94.7)	3690 (92.1)	3865 (92.1)	3691 (90.4)	15257 (92.3)
Small peripheral regions	135 (3.2)	237 (5.9)	218 (5.2)	262 (6.4)	853 (5.2)
Metropolis	89 (2.1)	80 (2.0)	113 (2.7)	130 (3.2)	413 (2.5)

n (%): weighted count and proportion for each study factors
